# Long noncoding RNA *lnc_217* regulates hepatic lipid metabolism by modulating lipogenesis and fatty acid oxidation

**DOI:** 10.7555/JBR.37.20230075

**Published:** 2023-11-15

**Authors:** Xiaoqing Yuan, Yawei Liu, Xule Yang, Yun Huang, Xuan Shen, Hui Liang, Hongwen Zhou, Qian Wang, Xu Zhang, John Zhong Li

**Affiliations:** 1 The Key Laboratory of Rare Metabolic Disease, Department of Biochemistry and Molecular Biology, the Key Laboratory of Human Functional Genomics of Jiangsu Province, Key Laboratory of Targeted Intervention of Cardiovascular Disease, Collaborative Innovation Center for Cardiovascular Disease Translational Medicine, Nanjing Medical University, Nanjing, Jiangsu 211166, China; 2 Department of General Surgery, the First Affiliated Hospital of Nanjing Medical University, Nanjing, Jiangsu 210029, China; 3 Department of Endocrinology, the First Affiliated Hospital of Nanjing Medical University, Nanjing, Jiangsu 210029, China

**Keywords:** NAFLD, lncRNA, *de novo* synthesis, β-oxidation

## Abstract

Nonalcoholic fatty liver disease (NAFLD) is considered a major health epidemic with an estimated 32.4% worldwide prevalence. No drugs have yet been approved and therapeutic nodes remain a major unmet need. Long noncoding RNAs are emerging as an important class of novel regulators influencing multiple biological processes and the pathogenesis of NAFLD. Herein, we described a novel long noncoding RNA, *lnc_217*, which was liver enriched and upregulated in high-fat diet-fed mice, and a genetic animal model of NAFLD. We found that liver specific knockdown of *lnc_217* was resistant to high-fat diet-induced hepatic lipid accumulation and decreased serum lipid in mice. Mechanistically, we demonstrated that knockdown of *lnc_217* not only decreased *de novo* lipogenesis by inhibiting sterol regulatory element binding protein-1c cleavage but also increased fatty acid β-oxidation through activation of peroxisome proliferator-activated receptor α and carnitine palmitoyltransferase-1α. Taken together, we conclude that *lnc_217* may be a novel regulator of hepatic lipid metabolism and a potential therapeutic target for the treatment of hepatic steatosis and NAFLD-related metabolic disorders.

## Introduction

Nonalcoholic fatty liver disease (NAFLD) has emerged as a significant public health problem, paralleling the dramatic escalation in the global prevalence of obesity. NAFLD affects about 32.4% of people worldwide, with prevalence rates varying from 13% in Africa to 42% in Southeast Asia ^[ 1] ^. By 2030, the number of NAFLD cases in China is predicted to increase to 314.58 million, which is the fastest growth in the prevalence of NAFLD globally ^[ 2] ^. Currently, there are no licensed drug treatments available for NAFLD, although there have been many drugs in the pipeline that are reckoned as good candidates to cure NAFLD/nonalcoholic steatohepatitis ^[ 3] ^. There is an unmet need to use natural products to cure or alleviate NAFLD by physicians ^[ 4] ^.

The pathophysiology of NAFLD has not been elucidated. However, it is known to develop when the influx of lipids traveling into the liver ( *i.e.*, fatty acid uptake and *de novo* lipogenesis [DNL]) exceeds hepatic lipid disposal ( *i.e.*, mitochondrial fatty acid oxidation and exportation as a component of very low-density lipoprotein [VLDL] particles). Studies reported that hepatic DNL made up about one-third of the total triglyceride content in the liver of NAFLD patients with hyperinsulinemia ^[ 5– 6] ^. Sterol regulatory element binding protein-1c (SREBP-1c) is a key lipogenic transcription factor, which directly activates the expression of more than 30 proteins, including fatty acid synthase (FAS) and acetyl-CoA carboxylase (ACC), and is involved in fatty acid and triglyceride synthesis ^[ 7] ^. Several lines of evidence implied that hyperinsulinemia, which predominated in the insulin-resistant state, stimulated lipogenesis by activating SREBP-1c, causing NAFLD in humans and animal models ^[ 8– 9] ^. During starvation, the body is powered mainly by adenosine triphosphate through β-oxidation of fatty acids, and this process was found to be modulated by carnitine palmitoyltransferase-1A (CPT-1A), a rate-limiting enzyme playing a crucial role in controlling fatty acyl-coA shuttle from the cytosol into mitochondrial matrix, where fatty acyl-coA underwent β-oxidation to produce energy in the form of adenosine triphosphate ^[ 10] ^. Moreover, transcription factor peroxisome proliferator-activated receptor α (PPARα) was reported to promote the transcription of CPT-1 to activate fatty acid β-oxidation and the generation of ketone bodies ^[ 11] ^. During starvation or fasting, one study showed that the defects in the *Pparα* gene led to hepatic steatosis and the development of hypoketosis and hypoglycemia in mice ^[ 12] ^. Of note, in previous studies, SREBP-1c expression decreased in PPARα-null mice, compared with wild-type mice ^[ 13] ^, and PPARα agonists enhanced the activity of the SREBP-1c promoter through direct binding with its DR1 motif ^[ 14] ^. Others reported that malonyl-CoA produced by ACC inhibited the activity of CPT-1, and thereby decreased the rate of β-oxidation by reducing fatty acid transport to mitochondria ^[ 15– 16] ^.

Long non-coding RNAs (lncRNAs) are a type of RNA, generally defined as transcripts more than 200 nucleotides that are not translated into protein ^[ 17– 18] ^. Similar to protein-coding genes, many lncRNAs are restricted in the tissue distribution ^[ 19] ^. Recent studies have demonstrated that lncRNAs are essential for fatty acid biosynthesis, oxidation, and VLDL secretion in the liver ^[ 20– 22] ^. For example, *lncHR1* was reported to be involved in the activation of SREBP-1c to regulate hepatic fatty acid synthesis ^[ 23] ^. Up-regulated long non-coding RNA ( *HULC*) was found to target PPARα to regulate lipid deposition in hepatocellular carcinoma ^[ 24] ^. Therefore, to further identify new lncRNAs that regulate hepatic lipid metabolism is of great importance.

To explore new non-coding RNA related to liver lipid metabolism, we analyzed an existing RNA-seq dataset (GSE157482) from the livers of high-fat diet (HFD) and normal diet (ND) mice and identified a novel lipid-induced lncRNA, *lnc_217*. To investigate its function, we performed gain and loss of function experiments to manipulate *lnc_217* expression levels *in vivo* and *in vitro*, based on adenovirus transduction and plasmid transfection, to clarify the role of a novel lncRNA in hepatic fatty acid metabolism, and to provide a new potential therapeutic target for the treatment of hepatic steatosis and NAFLD-related metabolic disorders.

## Materials and methods

### Experimental animals

C57BL/6J mice used in the current study were purchased from the Model Animal Research Center of Nanjing University (MARC, Nanjing, Jiangsu, China). All of the mice were maintained in a 12-h light/dark cycle at 25 ℃ with free access to rodent chow and water in the animal facility (specific-pathogen free) of Nanjing Medical University. All the procedures followed the guidelines for the care and use of animals established by Nanjing Medical University (IACUC-1601211).

### Cell culture and transfection

293A and Hepa1-6 cells were purchased from the American Type Culture Collection (ATCC, Manassas, VA, USA) and maintained in high-glucose DMEM (Life Technologies, Gaithersburg, MD, USA) containing 10% FBS (Gibco, Waltham, MA, USA) and 1% penicillin-streptomycin. The full-length *lnc_217* expression vectors with Myc-Tag were amplified by PCR from C57BL/6J mouse liver cDNA. The specific primers were listed as follows: *lnc_217*-F: 5′-GTGCCAGACTACGCAGGATCCATGCTCATCATCTTGCCTCTGGG-3′; *lnc_217*-R: 5′-CGCCTCGAGAAGCTTGGATCCGTGGCTTTCCTGTGCACGTGTTG-3′; *lnc_217*-T-R: 5′-CGCCTCGAGAAGCTTGGATCCTGTGGCTTTCCTGTGCACGTGTTG-3′; *lnc_217*-TT-R: 5′-CGCCTCGAGAAGCTTGGATCCTTGTGGCTTTCCTGTGCACGTGTTG-3′. Lipofectamine 2000 (Invitrogen, Carlsbad, CA, USA) was used for transient transfection in 293A and Hepa1-6 cells according to the manufacturer's instructions.

### Adenovirus-mediated knockdown of *lnc_217* in mice

Adenovirus of Ad-scramble or Ad-sh *lnc_217* was generated by the AdEasy system (sh *lnc_217* target sequences: 5′-CTAAGCATGACAAATCACT-3′, 3′- AGTGATTTGTCATGCTTAG-5′) and injected ( *n* = 6/group, 2.5 × 10 ^11^ particles/mouse) *via* tail veins of 8–10-week-old C57BL/6J mice. After injection, mice were given either an ND or an HFD (60% kcal from fat, D12492, Research Diets, New Brunswick, NJ, USA) for seven days. After a 16-h fast, the mice were then euthanatized, and the liver and blood were harvested.

### Mouse primary hepatocyte isolation and treatment

Mouse primary hepatocytes were isolated in a previously mentioned manner ^[ 21] ^. Hepatocytes were cultured in a low glucose medium (Gibco) with 10% FBS supplement in 6-well plates at a density of 4 × 10 ^5^ cells per well. Adenovirus was applied to primary hepatocytes (6 × 10 ^9^ particles/well) for 6 h. Following a 16-h incubation period with or without 200 μmol/L oleic acid, cells were harvested.

### Real-time reverse transcription PCR (RT-qPCR)

The livers were immediately frozen in liquid nitrogen, and RNA was isolated using Trizol (Invitrogen), according to the manufacturer's instructions. The cDNA synthesis and quantitative PCR with the indicated primers ( ***Supplementary Table 1 ***, available online) were performed as described. RNA expression levels were normalized to *Actb* as the internal control and to the control group using the 2 ^−ΔΔCt^ method, calculated as arbitrary units, and represented as mean ± standard deviation. The data of the expression levels of *lnc_217* in different tissues of C57BL/6J mice were expressed as mean.

### Western blotting

Fresh tissues or cells were harvested and lysed in RIPA buffer supplemented with a complete protease inhibitor cocktail (Roche Diagnostics Deutschland GmbH, Mannheim, Germany), and their protein concentration was subsequently determined by the BCA assay. Then, SDS-PAGE and wet transfer were performed. The membranes were blocked with 5% milk and then incubated with the indicated primary antibodies followed by HRP-conjugated secondary antibody. Proteins were visualized using Super-Signal ECL (Biotanon, Shanghai, China). The following antigens were targeted by the use of antibodies: anti-SREBP-1c (1∶1000; Cat. #ab28481, Abcam, Cambridge, UK), anti-PPAR (1∶1000; Cat. #ab24509, Abcam), anti-CPT1α (1∶1000; Cat. #CPT1L12-A, Alpha Diagnostic, San Antonio, TX, USA), anti-UCP2 (1∶1000; Cat. #ab67241, Abcam), and Calnexin (1∶3000; Cat. #ADI-SPA-860, ENZO Life Sciences, Farmingdale, NY, USA). Protein levels were normalized to calnexin as the internal control and to the control group.

### Liver and plasma lipids analysis

Liver lipids were extracted from 100–200 mg of frozen liver samples using the Folch and Lees' method. Triglyceride, total cholesterol and free fatty acid were measured using enzymatic kits (Wako, Richmond, VA, USA) and normalized to sample weight. Plasma triglyceride, total cholesterol and free fatty acid were measured using the same enzymatic assays.

### Oil red O staining

Liver sections were embedded in liquid nitrogen and transversely sectioned into 10-μm frozen sections for oil red O staining. Stained sections were analyzed using ImageJ software (NIH, http://rsb.info.nih.gov/ij/). We opened the raw image and split it into three color-channels with "Split channels". The blue channel, which highlighted its raw oil-red staining, was chosen to set an appropriate threshold. Threshold values were determined empirically by selecting a setting that gave the most accurate binary image for a subset of randomly selected photomicrographs with varying peptides densities. Then, the resulting image was measured by "Area fraction" measurements. Six slices were analyzed per group.

### 5′ and 3′ rapid amplification of cDNA ends (RACE)

To identify the full-length sequence of *lnc_217*, the GeneRacer Kit (Invitrogen) was used to perform 5′ and 3′ cDNA terminal rapid amplification tests according to the manufacturer's protocol. For the *lnc_217* RACE assay, sequences in the mouse genome were retrieved from the NCBI database using the Basic Local Alignment Search Tool, and confirmed by RT-PCR using forward (5′-GGTTAGGACCCGTCAG-3′) and reverse (5′-CAGTGAGCGAGTCTATTT-3′) primers and sequencing. From the sequencing results, 5′ and 3′ RACE primers were designed. The sequences of gene-specific primers were 5′-ACTCATCATCTTGCCTCTGGGAATC-3′ (5′ RACE) and 5′-GCCTCACTGTTTTGCCTGGTG-3′ (3′ RACE).

### RNA fluorescence *in situ* hybridization (RNA-FISH)

*Lnc_217* was detected by RNA-FISH in mouse hepatocytes. Cell slides fixed with 4% paraformaldehyde were treated with several buffers in the RNA-FISH Kit (Ri-boBio, Guangzhou, China). After denaturation at 37 ℃ for 5 min, the probe mixture was hybridized overnight in cell slides at 37 ℃ in the dark. CY3-labeled Locked Nucleic Acid probes targeted *lnc_217*. After washing, the signal was observed under a confocal microscope.

### Statistical analysis

Statistical analysis was performed by GraphPad Prism 7. Results were presented as the mean and standard deviation of at least three independent experiments as indicated in the figure legends. A two-tailed Student's *t*-test was used to calculate the statistical significance of different groups. A two-way ANOVA was used for statistical analysis in ***Supplementary Fig. 2*** (available online). Statistical results were considered significant when *P* < 0.05.

## Results

### Identification of a novel lncRNA implicated in hepatic lipid metabolism

**Figure 1:**
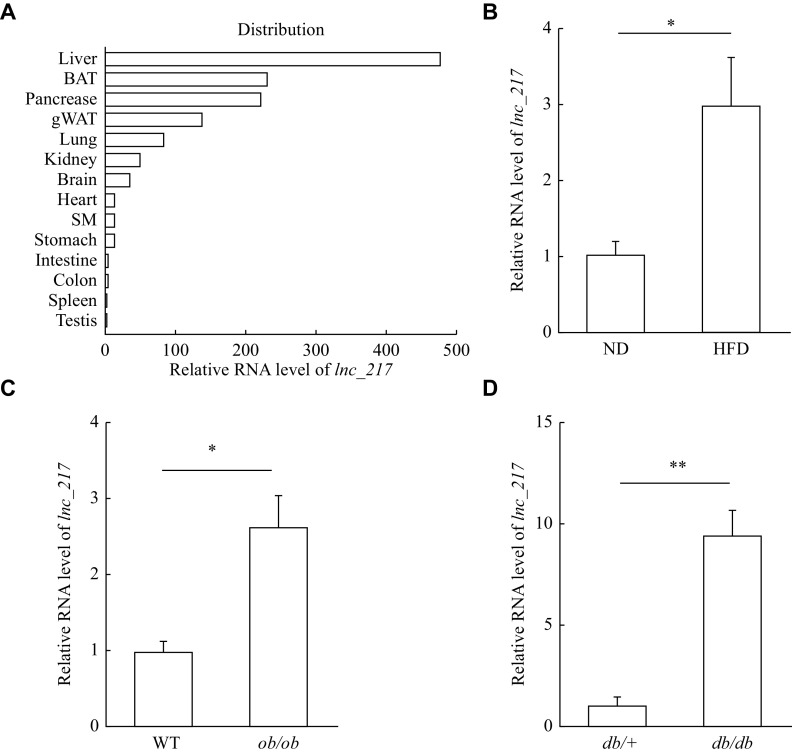
Identification of a novel long non-coding RNA (lncRNA) implicated in hepatic lipid metabolism.

To identify novel lncRNAs potentially involved in hepatic lipid metabolism, we analyzed a previously published RNA-seq dataset (GSE157482) from the livers of HFD and ND mice ^[ 21] ^. Among the altered lncRNAs, we found that the expression of *lnc_217* was enriched in the mouse liver ( ***Fig. 1A***) and significantly increased in the liver of the HFD mice ( ***Fig. 1B***). In addition to the HFD mouse model, *ob/ob* and *db/db* mice are important genetic mouse models of NAFLD. The hepatic expression levels of *lnc_217* were significantly increased in these two NAFLD mouse models ( ***Fig. 1C***– ***1D***). Taken together, *lnc_217* is regulated by nutritional status and may be correlated with the pathogenesis of hepatic steatosis.

### Prediction and verification of the encoding ability of *lnc_217*

**Figure 2:**
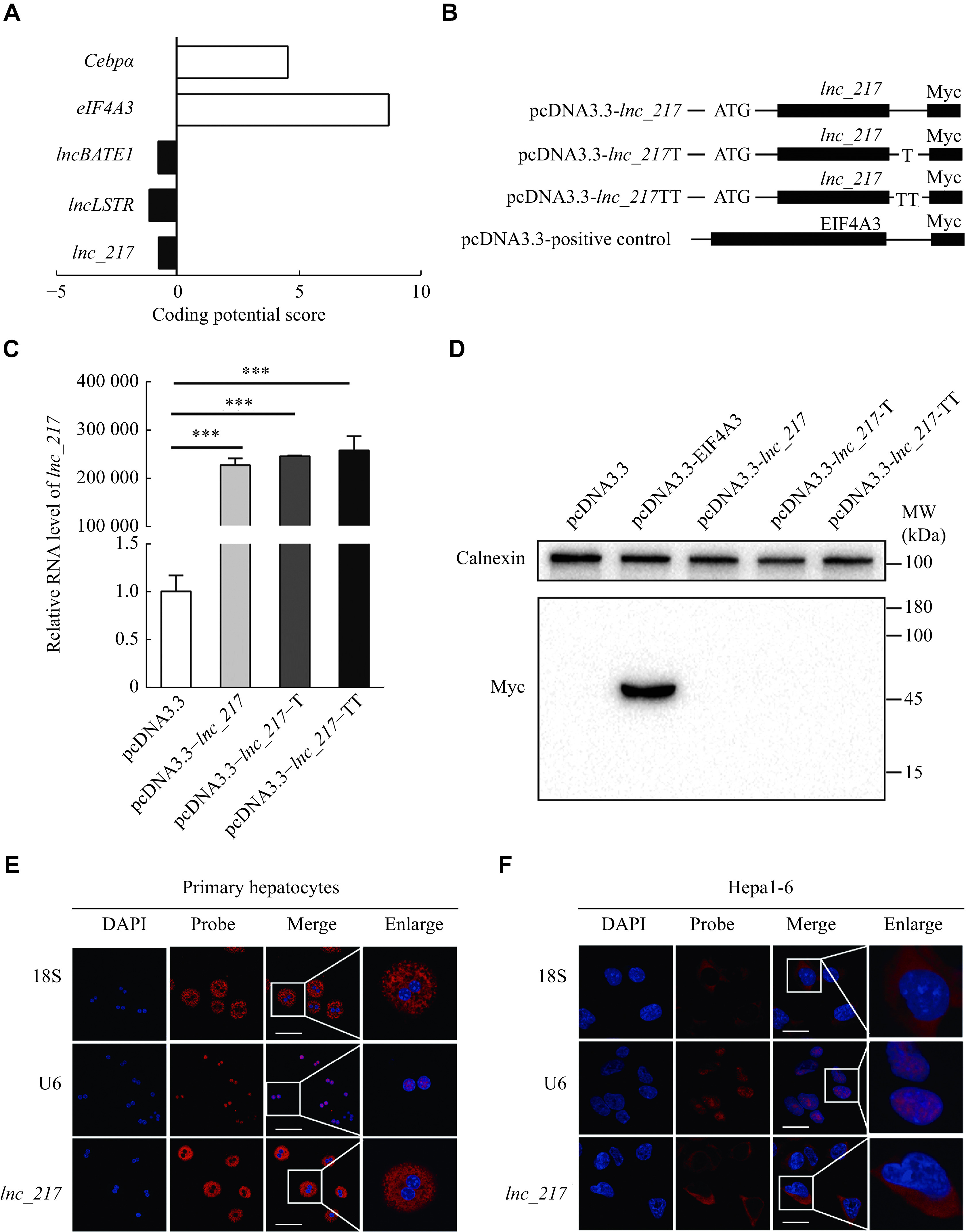
Characterization of the non-coding property and sub-cellular location of *lnc_217*.

The results of the RACE assay revealed a full-length of 2978 nt for *lnc_217* located on chromosome 7 of mice ( ***Supplementary Fig. 1A*** and ***1B ***, available online), which was highly conserved from mice to humans. According to the prediction of the Coding Potential Calculator online prediction system ( http://cpc.gao-lab.org/), *lnc_217* lacks coding potential, similar to known lncRNAs, such as *lncLSTR* and *lncBATE1* ( ***Fig. 2A***). In addition, the full-length *lnc_217* expression vectors with Myc-Tag were constructed and transfected into 293A cells to further elucidate protein coding potential ( ***Fig. 2B***). All the vectors were transcribed but failed to produce protein ( ***Fig. 2C*** and ***2D***). These results confirmed that *lnc_217* lacked the ability to encode proteins. RNA-FISH analysis further revealed that *lnc_217* was enriched in the cytosol fraction of primary hepatocytes and Hepa1-6 hepatocytes with a similar expression pattern to 18S ribosomal RNA, a positive control marker for the cytosol fraction ( ***Fig. 2E*** and ***2F***). These results suggest that *lnc_217* is a lncRNA localized in the cytosol without the protein coding ability.

### Liver-specific knockdown of *lnc_217* reduced hepatic lipid accumulation and hyperlipidaemia in HFD-fed mice

**Figure 3:**
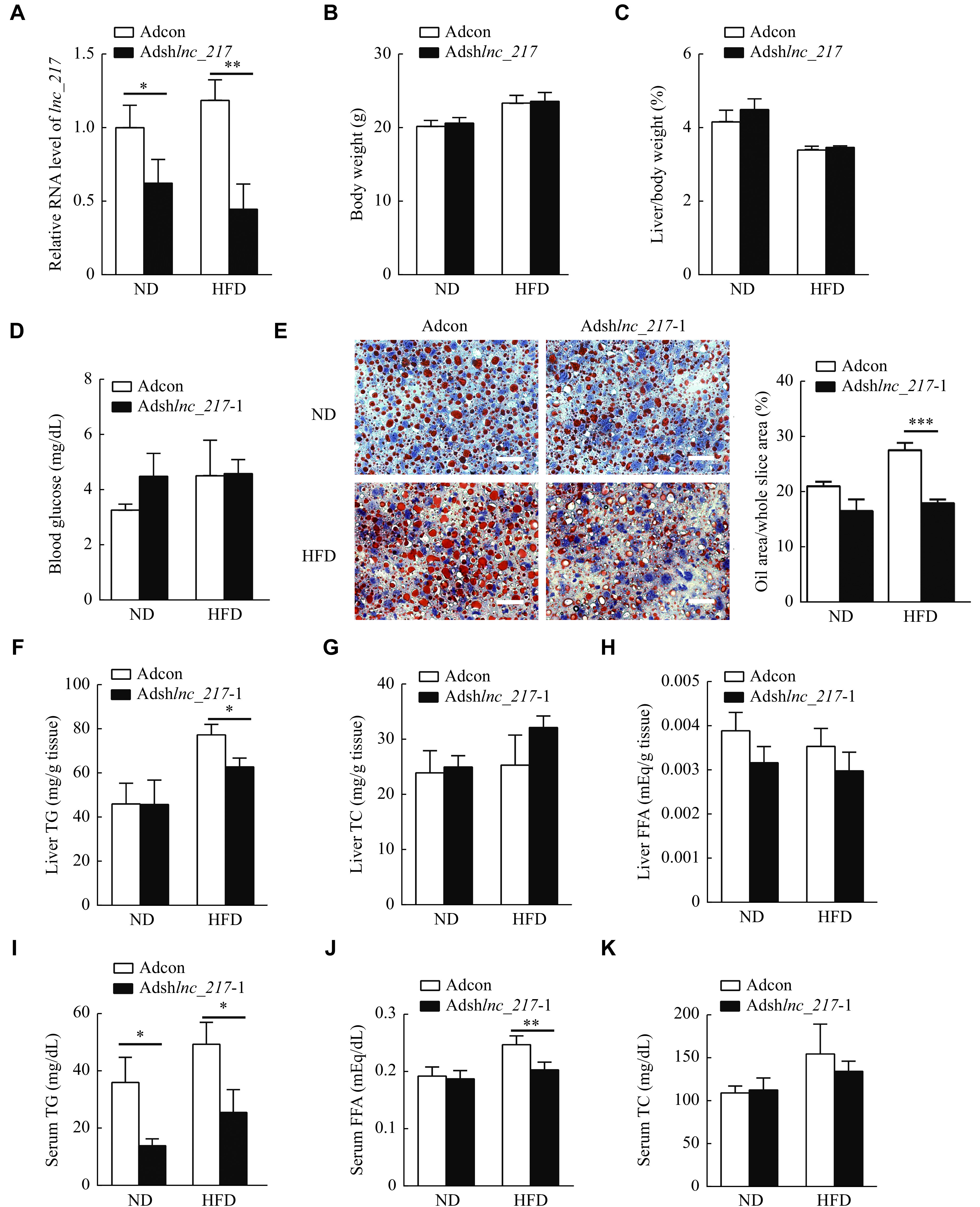
Knockdown of *lnc_217* reduced hepatic lipid accumulation and hyperlidiaemai in high-fat diet fed (HFD) mice.

To investigate physiological functions of *lnc_217* in the hepatic lipid metabolism, we generated liver-specific *lnc_217*-knockdown mice by tail vein injection with adenoviral-mediated shRNA ( ***Fig. 3A***) and then fed the mice an HFD. As shown in ***Fig. 3B***, there was no significant difference in the body weight ( ***Fig. 3B***) or the liver index ( ***Fig. 3C***) between the *lnc_217* knockdown group and the control group. There was also no statistically significant difference in the blood glucose levels between the two groups of mice ( ***Fig. 3D***). These findings suggest that liver-specific *lnc_217* knockdown may not cause disturbances in the glucose metabolism.

To further evaluate biological functions of *lnc_217* in the hepatic lipid metabolism, oil red O staining of mouse liver sections was performed. The results showed that the knockdown of *lnc_217* significantly inhibited HFD-induced lipid accumulation in the liver ( ***Fig. 3E***). Consistently, the hepatic triglyceride level was significantly reduced in liver-specific *lnc_217* knockdown mice, compared with that in the control group with HFD challenge ( ***Fig. 3F***). However, we found that the knockdown of *lnc_217* did not change hepatic cholesterol as well as free fatty acid levels ( ***Fig. 3G*** and ***3H***). The results of further biochemical analysis of mouse serum revealed that the knockdown of *lnc_217* significantly decreased serum triglyceride and free fatty acid levels after HFD feeding ( ***Fig. 3I*** and ***3J***), without affecting serum total cholesterol levels ( ***Fig. 3K***). Taken together, the knockdown of *lnc_217* ameliorates HFD-induced liver lipid accumulation and hyperlipidemia in mice.

### Liver-specific knockdown of *lnc_217* decreased liver fatty acid synthesis and increased β-oxidation

Hepatic lipid homeostasis is mainly coordinated by DNL, fatty acid β-oxidation, and VLDL secretion. We first performed a VLDL-triglyceride secretion assay by injection of tyloxapol to verify the regulatory role of *lnc_217* on hepatic lipid secretion *in vivo.* As shown in ***Supplementary Fig. 2A*** (available online), the knockdown of *lnc_217* did not alter VLDL secretion.

**Figure 4:**
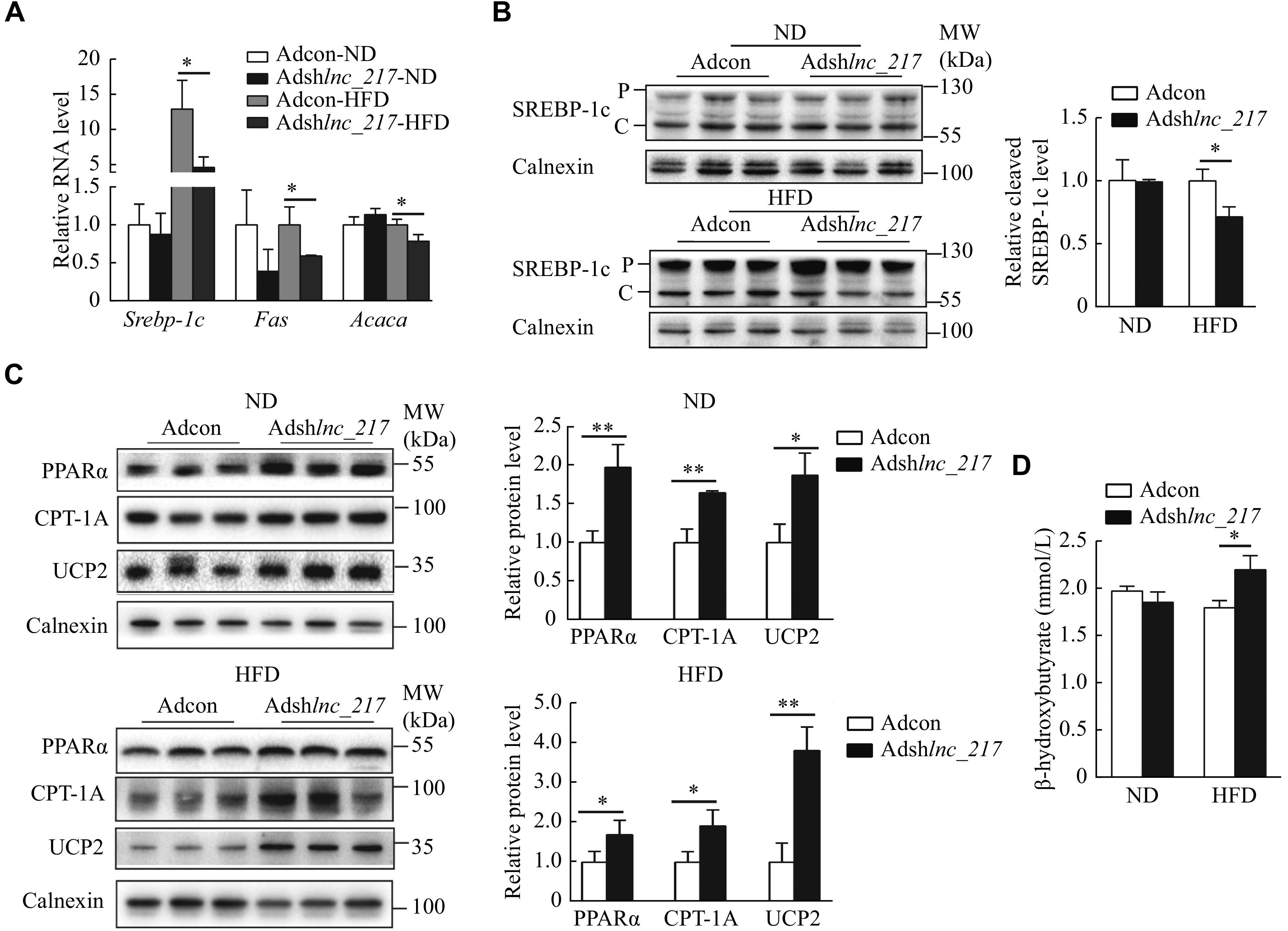
Liver-specific knockdown of *lnc_217*decreased hepatic lipogenesis and increased fatty acid β-oxiation in mice.

Importantly, the RT-qPCR results showed that liver-specific knockdown of *lnc_217* significantly repressed hepatic expression of key genes involved in DNL, such as *Srebp-1c*, *Fas* (the gene encoding tumor necrosis factor receptor superfamily member 6), and *Acaca* (the gene encoding acetyl-CoA carboxylase 1) ( ***Fig. 4A***). We further measured the protein level of SREBP-1c in the livers of these mice and found that manipulation of *lnc_217* did not change the precursor level of SREBP-1c; however, the cleavage form of SREBP-1c protein level significantly decreased in the liver of *lnc_217* knockdown mice with an HFD ( ***Fig. 4B***). These results suggest that the knockdown of *lnc_217* decreases SREBP-1c expression and cleavage to inhibit hepatic fatty acid synthesis.

Furthermore, we observed that the protein levels of PPARα, CPT-1A and UCP2 were significantly induced in the liver of mice with the depletion of *lnc_217* ( ***Fig. 4C***). Consistently, the serum level of β-hydroxybutyrate, a major ketone body generated by fatty acid β-oxidation in the liver, was significantly increased in the *lnc_217* knockdown mice after HFD feeding ( ***Fig. 4D***). These results suggest that the knockdown of *lnc_217* promotes fatty acid β-oxidation in the liver.

### Knockdown of *lnc_217* decreased fatty acid synthesis and increased β-oxidation in primary hepatocytes

**Figure 5:**
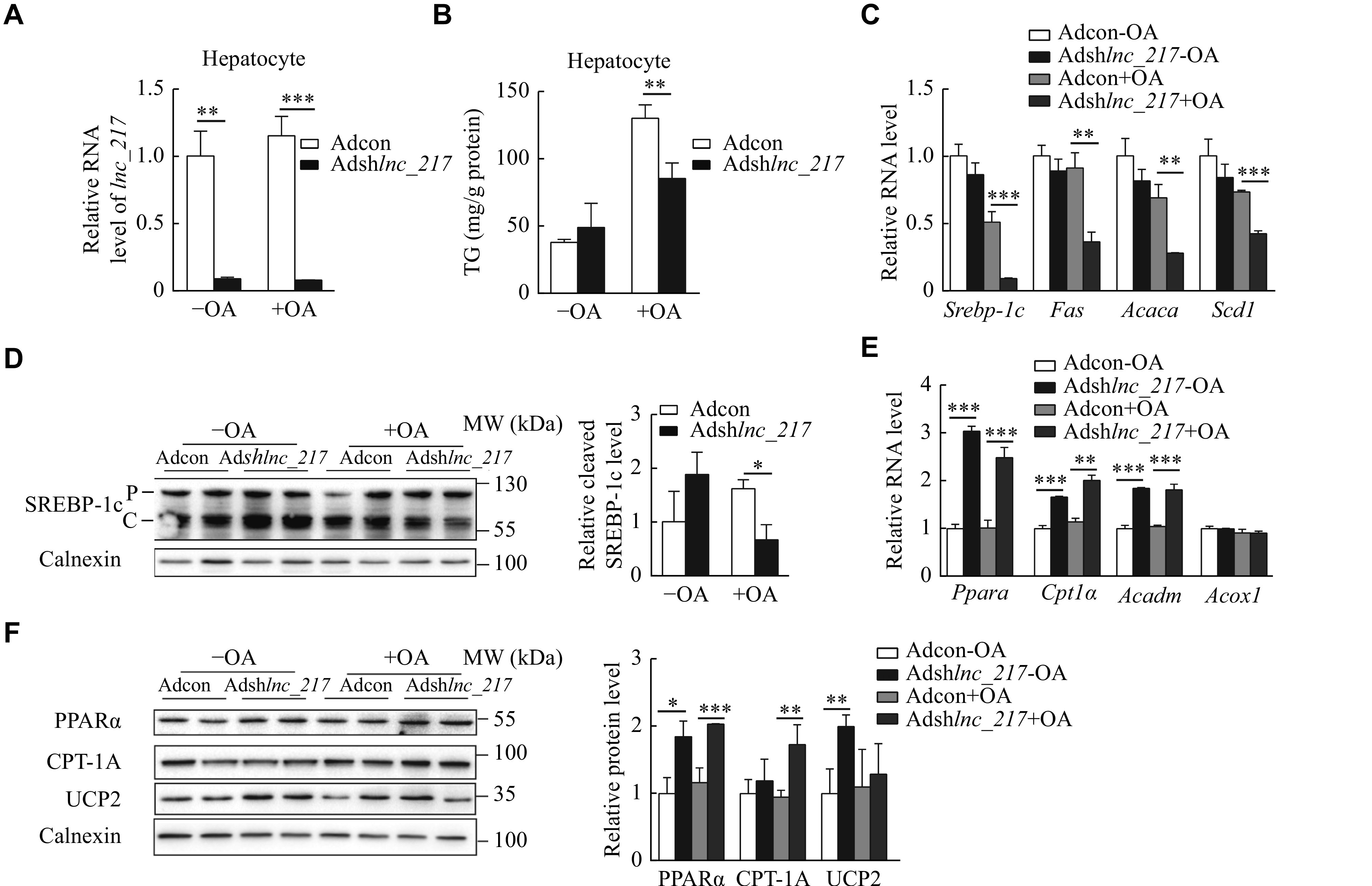
Knockdown of *lnc_217* decreased lipogenesis and increased fatty acid β-oxidation in primary hepatocytes.

To confirm that the decrease in liver triglyceride in the *lnc_217* knockdown mice was cell autonomous, we further investigated biological consequences of the *lnc_217* knockdown on the lipid metabolism in primary hepatocytes infected with Ad-sh *lnc_217* ( ***Fig. 5A***). As shown in ***Fig. 5B***, the triglyceride levels were reduced by about 40% in hepatocytes after knockdown of *lnc_217* in the presence of oleic acid. Consistent with the observations in mice liver, the mRNA levels of genes involved in lipogenesis, such as *Srebp-1c*, *Fas*, *Acc1*, and *Scd1* (the gene encoding acyl-CoA desaturase 1), markedly declined in *lnc_217*-knockdown hepatocytes with oleic acid treatment ( ***Fig. 5C***). The cleavage of SREBP-1c was also reduced ( ***Fig. 5D***). In addition, the expressions of PPARα and CPT-1A were significantly upregulated in both mRNA ( ***Fig. 5E***) and protein levels ( ***Fig. 5F***). These data suggest that the reduced lipid contents in *lnc_217* knockdown hepatocytes result directly from a combination effect of the reduced DNL and the increased fatty acid β-oxidation.

### Overexpression of *lnc_217* increased lipogenesis and decreased fatty acid β-oxidation in Hepa1-6 cells

**Figure 6:**
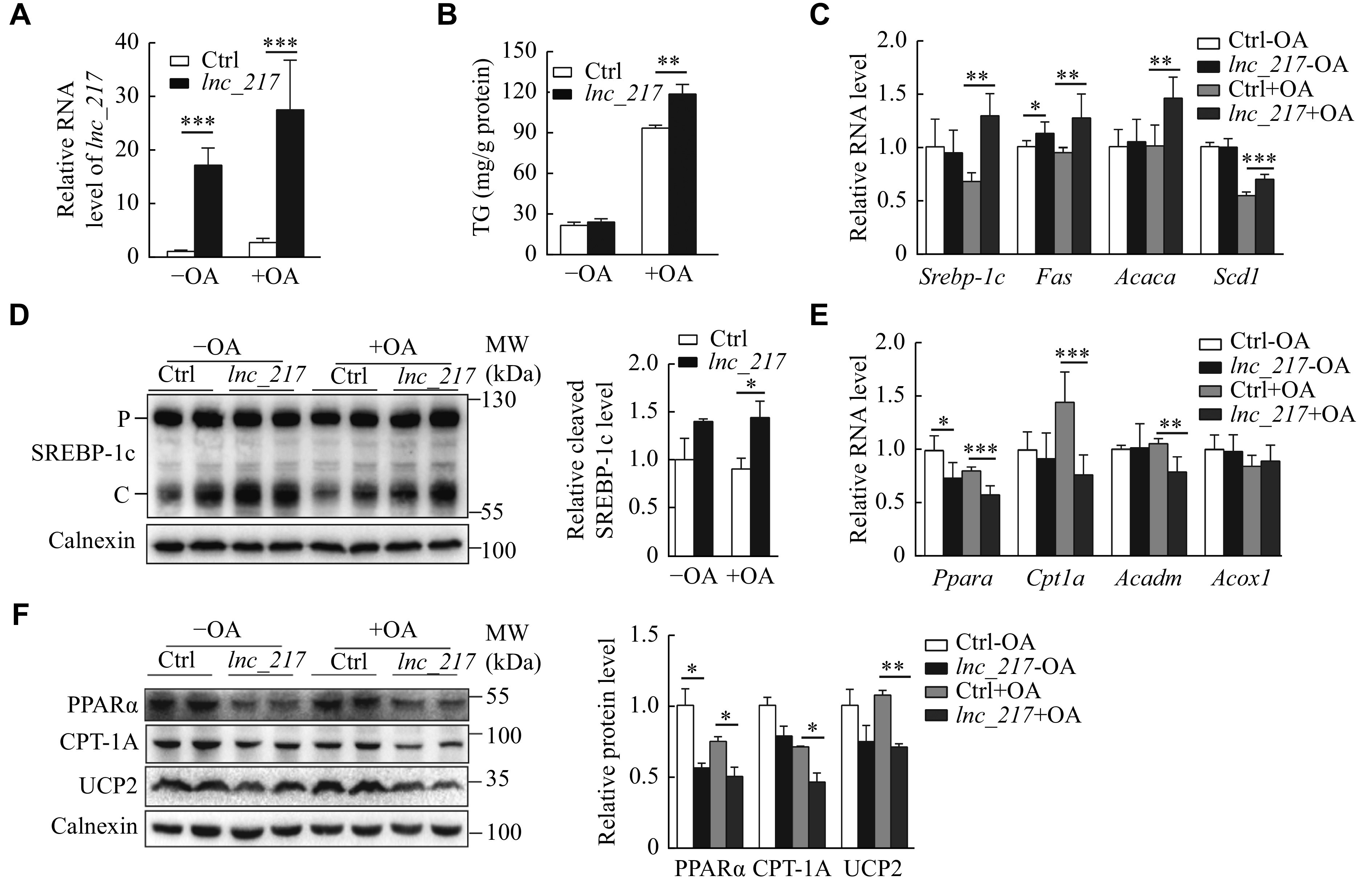
Overexpression of *lnc_217* increased lipogenesis and decreased fatty acid β-oxidation in Hepa1-6 cells.

Apart from the knockdown experiments, we overexpressed *lnc_217* in Hepa1-6 cells. In agreement with the knockdown result, the overexpression of *lnc_217* resulted in a significant accumulation of triglycerides following oleic acid treatment ( ***Fig. 6A*** and ***6B***). Furthermore, the mRNA levels of lipogenic genes, including *Srebp-1c*, *Fas*, *Acc1* and *Scd1*, markedly increased in the *lnc_217*-overexpressed cells ( ***Fig. 6C***), and the cleavage of SREBP-1c was also elevated ( ***Fig. 6D***). In addition, the expression levels of fatty acid oxidation genes ( *Ppara* and *Cpt1a*) were significantly downregulated ( ***Fig. 6E*** and ***6F***). These data suggest that the overexpression of *lnc_217* may directly induce hepatic steatosis. Based on the results from the knockdown and overexpression experiments, we concluded that *lnc_217* regulated the hepatic lipid metabolism by modulating lipogenesis and fatty acid oxidation.

## Discussion

Given the prevalence of NAFLD, its associated complications, and its lack of effective treatment options, the understanding of hepatic fatty acid metabolism is a vital research priority as we strive to find effective prevention or therapeutic strategies for NAFLD ^[ 25] ^. NAFLD stems from a disequilibrium between hepatic fatty acid influx and disposal. The choice of interventional targets is challenging due to the existence of feedback mechanisms among fatty acid absorption, esterification, oxidation and secretion ^[ 26] ^. LncRNAs are emerging as an important class of novel regulators influencing multiple biological processes and the pathogenesis of metabolic diseases ^[ 27– 28] ^. In the current study, we demonstrated that a novel lncRNA, *lnc_217*, may be a potential therapeutic target for NAFLD, as it effectively balanced the low synthesis and high consumption of fatty acids in the liver.

Various conditions lead to hepatic steatosis in the liver. In the current study, experiments with primary hepatocytes could exclude the uptake of free fatty acids from serum by hepatocytes. A tyloxapol-induced VLDL secretion experiment ruled out triglyceride secretion. It is well known that enhanced activation of SREBP-1c is closely associated with the development of hepatic steatosis and dyslipidemia. The suppression of SREBP-1c cleavage has been proven to be an effective approach to improve fatty liver in mice. The knockdown of *lnc_217* reduces cleavage of SREBP-1c, as well as the transcription of its target genes. Thus, the knockdown of *lnc_217* decreases hepatic steatosis by inhibiting DNL of fatty acids. On the other hand, *lnc_217* knockdown increases the levels of PPARα, CPT-1A and UCP2, which are key regulators of β-oxidation, both *in vitro* and *in vivo*. In agreement with this observation, *lnc_217* knockdown increases the content of ketone bodies in the blood. It is well known that ACC plays a crucial role in fatty acid oxidation by converting acetyl-CoA into malonyl-CoA, which in turn inhibits the CPT-1 activity and fatty acid transportation into mitochondria, thus reducing oxidation rates. Therefore, the reduced ACC levels may, at least in part, account for the increased fatty acid oxidation and energy expenditure in *lnc_217* knockdown mice.

The function of lncRNA is determined by its subcellular localization. In the cytoplasm, lncRNAs regulate the stability and translation of target mRNAs either directly or indirectly by utilizing microRNAs ^[ 29] ^. In gastro-carcinoma cells, MSC-induced lncRNAs *HCP5* and *MACC1-AS* exerted anti-tumour effects by inhibiting the ability of microRNA to activate CPT-1 expression and β-oxidation ^[ 22, 30] ^. Within the nucleus, lncRNAs play a role in transcriptional regulation in some ways. Human-specific *lncHR1* is located in the nucleus and is involved in the transcriptional inhibition of *SREBP-1c* ^[ 23] ^. Several nuclear-expressed lncRNAs, such as *MALAT1*, do not affect *SREBP-1c* transcription but alter protein shearing ^[ 31] ^. Similar to gene expression, subcellular localization of lncRNAs is a dynamic process ^[ 32] ^. In the current study, we found that *lnc_217* was distributed mainly in the cytosol. The knockdown of *lnc_217* reduced SREBP-1c transcription and cleavage. In addition, genes involved in β-oxidation were significantly elevated at the transcriptional level. This finding implies that *lnc_217* may be involved in the stability and translation of target mRNAs or that the subcellular localization of *lnc_217* may be altered. However, the specific mechanism remains unclear. In future studies, we will search for direct target genes of *lnc_217* through RNA pull-down and RNA-binding protein immunoprecipitation experiments to better understand the molecular mechanism of *lnc_217* in regulating hepatic lipid metabolism and the development of NAFLD.

Taken together, the current study provides the first evidence that *lnc_217* is closely involved in the regulation of hepatic lipogenesis and β-oxidation. The expression of *lnc_217* is increased in a variety of animal models of fatty liver, suggesting that *lnc_217* is likely involved in the progression of NAFLD. The knockdown of *lnc_217* ameliorated HFD-induced hepatic steatosis and hyperlipidemia. The dual effects of *lnc_217* on fatty acid synthesis and β-oxidation make *lnc_217* an appropriate therapeutic target for NAFLD. Therefore, we have revealed a new molecular mechanism of lncRNA regulation of the hepatic fatty acid metabolism, which may pave the way for clinical treatments for NAFLD and dyslipidemia.
